# Supplementary material for: Using Design of Experiments to Optimize a Screening Analytical Methodology Based on Solid-Phase Microextraction/Gas Chromatography for the Determination of Volatile Methylsiloxanes in Water
Source: Molecules. 2021 Jun 5;26(11):3429. doi: 10.3390/molecules26113429 (PMC8201336; doi:10.3390/molecules26113429)
Supplement: Supplementary file 1 [file molecules-26-03429-s001.zip › molecules-1234873-supplementary.pdf]

Supporting Information

Using Design of Experiments to optimize a screening analytical methodology based on Solid-Phase Microextraction/Gas Chromatography for the determination of volatile methylsiloxanes in water

Fábio Bernardo[1], Providencia González-Hernández[2], Nuno Ratola[1], Verónica Pino[2,3], Arminda Alves[1], Vera Homem[1,*]

[1]LEPABE – Laboratory for Process Engineering, Environment, Biotechnology and Energy, Faculty of Engineering, University of Porto, Rua Dr. Roberto Frias, 4200-465 Porto, Portugal

[2]Departamento de Química, Unidad Departamental de Química Analítica, Universidad de La Laguna (ULL), La Laguna, Tenerife, 38206 Spain

[3]University Institute of Tropical Diseases and Public Health, Universidad de La Laguna (ULL), La Laguna, Tenerife, 38206 Spain

Number of Pages: 6

Number of Tables: 5

Number of Figures: 2

Table S1. Factors and values for the proposed central composite design

X_i	Factor	-1.607 (-α)	-1 (-)	0	1 (+)	1.607 (+α)	Central Composite Design (CCD)
**1**	Ionic strength (%NaCl)	0	5	12	20	24	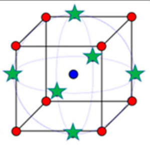
**2**	Extraction time (min)	3	11	25	39	48	
**3**	Desorption time (min)	1	3	6	10	12	
**4**	Extraction temperature (°C)	22	33	51	69	80	
**5**	Desorption temperature (°C)	201	210	225	240	249	

Table S2. Proposed central composite design

Run	Pattern	X_1	X_2	X_3	X_4	X_5
**1**	000(-α)0	12	25	6	22	225
**2**	++−−−	20	39	3	33	210
**3**	+−+−−	20	11	10	33	210
**4**	−−−−−	5	11	3	33	210
**5**	−++−−	5	39	10	33	210
**6**	+++−+	20	39	10	33	240
**7**	−+−−+	5	39	3	33	240
**8**	+−−−+	20	11	3	33	240
**9**	−−+−+	5	11	10	33	240
**10**	0000(-α)	12	25	6	51	201
**11**	00000	12	25	6	51	225
**12**	00000	12	25	6	51	225
**13**	(-α)0000	0	25	6	51	225
**14**	(+α)0000	24	25	6	51	225
**15**	00000	12	25	6	51	225
**16**	00000	12	25	6	51	225
**17**	00000	12	25	6	51	225
**18**	00(-α)00	12	25	1	51	225
**19**	00(+α)00	12	25	12	51	225
**20**	00000	12	25	6	51	225
**21**	0(-α)000	12	3	6	51	225
**22**	0(+α)000	12	48	6	51	225
**23**	0000(+α)	12	25	6	51	249
**24**	−+−+−	5	39	3	69	210
**25**	−−++−	5	11	10	69	210
**26**	++++−	20	39	10	69	210
**27**	+−−+−	20	11	3	69	210
**28**	+−+++	20	11	10	69	240
**29**	−−−++	5	11	3	69	240
**30**	−++++	5	39	10	69	240
**31**	++−++	20	39	3	69	240
**32**	000(+α)0	12	25	6	80	225

Table S3. Second-order polynomial equation obtained for each target compound and model suitability parameters for the response functions.

Compound	R^2	*F*-ratio	Prob > F	LOF Prob > F
**D3**	0.94	0.492	0.0003	0.806
$y = 15586 + 1734\,x_2 - 5654x_4 + \ 2475x_5$				
**L3**	0.97	8.999	<.0001	0.025
$y = 16053 - 2083\,x_1 + 1128x_2 - 7803x_4$				
**D4**	0.93	0.823	0.0009	0.615
$y = 34783 - 1762x_1 + 3441x_2 - 7568x_4 - 1951x_2^2 - 3282x_4^2$				
**L4**	0.84	7.445	0.0347	0.035
$y = 27384 + 2151x_2 - 3358x_4 - 2893x_4^2$				
**D5**	0.81	6.139	0.0733	0.049
$y = 36532 + 3634x_2 - 2247x_2^2 - 2884x_4^2$				
**M4Q**	0.70	0.662	0.3533	0.703
$y = 13352 + 2247x_1 + 2032x_1^2$				
**L5**	0.74	4.973	0.2135	0.070
$y = 38081 + 2514x_1 + 3577x_2$				
**D6**	0.80	4.522	0.0986	0.082
$y = 30897 + 3034x_2 - 2134x_2^2$				

Table S4. Results from the Student's t-test for the main, quadratic and interaction effects

	**Prob > |t|**							
	**D3**	**L3**	**D4**	**L4**	**D5**	**M4Q**	**L5**	**D6**
**Intercept**	**<0.0001**	**<0.0001**	**<0.0001**	**<0.0001**	**<0.0001**	**<0.0001**	**<0.0001**	**<0.0001**
**X1**	0.097	**0.002**	**0.091**	0.875	0.857	**0.023**	**0.057**	0.117
**X2**	**0.011**	**0.048**	**0.004**	**0.028**	**0.004**	0.826	**0.011**	**0.003**
**X3**	0.405	0.998	0.438	0.721	0.718	0.842	0.751	0.855
**X4**	**<0.0001**	**<0.0001**	**<0.0001**	**0.002**	0.114	0.113	0.574	0.202
**X5**	**0.001**	0.983	0.161	0.813	0.117	0.743	0.538	0.150
**X1X2**	0.952	0.585	0.679	0.418	0.400	0.690	0.478	0.425
**X1X3**	0.591	0.634	0.762	0.563	0.723	0.650	0.748	0.829
**X2X3**	0.136	0.180	0.355	0.294	0.349	0.626	0.487	0.364
**X1X4**	0.362	0.956	0.214	0.136	0.160	0.436	0.844	0.750
**X2X4**	0.553	0.900	0.625	0.959	0.468	0.893	0.781	0.756
**X3X4**	0.590	0.399	0.258	0.316	0.365	0.747	0.347	0.276
**X1X5**	0.890	0.260	0.665	0.448	0.406	0.765	0.363	0.548
**X2X5**	0.699	0.705	0.888	0.576	0.557	0.500	0.498	0.245
**X3X5**	0.903	0.864	0.652	0.301	0.311	0.332	0.318	0.164
**X4X5**	0.955	0.133	0.933	0.210	0.335	0.858	0.237	0.211
**X1²**	0.695	0.270	0.169	0.144	0.131	**0.045**	0.231	0.209
**X2²**	0.732	0.946	**0.075**	0.191	**0.054**	0.109	0.124	**0.025**
**X3²**	0.785	0.541	0.842	0.922	1.000	0.917	0.737	0.766
**X4²**	0.376	0.254	**0.007**	**0.007**	**0.018**	0.340	0.190	0.111
**X5²**	0.779	0.804	0.502	0.432	0.476	0.752	0.522	0.745

Blue – Prob > |t| below 0.05
Light Blue – Prob > |t| below 0.01

Table S5. Additional testing to prove the applicability of the proposed empirical model

	A (*Pattern:* (-α) (-α) (-α) (-α) (-α))				B (*Pattern:* (-α)++-+)				C (*Pattern:* ++(-α)-+)			
	**Area**	**±SD**	**Area Pred.**	**±SD**	**Area**	**±SD**	**Area Pred.**	**±SD**	**Area**	**±SD**	**Area Pred.**	**±SD**
**D3**	17262	1252	26048	11778	25804	3928	27427	6304	24072	870	25331	6320
**L3**	18363	1150	32056	10488	28472	2306	29690	5614	25959	2592	25425	5628
**D4**	20708	1515	34806	19667	41941	4294	48279	10526	42032	2319	48155	10553
**L4**	15259	1483	23147	17575	25282	3490	32171	9407	33625	3446	31926	9431
**D5**	16554	1187	27017	20679	33931	5102	41404	11023	39518	3933	41067	11096
**M4Q**	2243	914	15521	17662	6163	998	11987	9454	12941	414	16887	9477
**L5**	11983	527	28646	24448	21688	4745	37222	13086	44364	5197	40263	13118
**D6**	8410	318	21282	16294	21678	3077	33773	8721	34266	4076	31788	8743

	D (*Pattern: +0+-+*)				E (*Pattern:* (+α) (+α) (+α) (+α) (+α))			
	**Area**	**±SD**	**Area Pred.**	**±SD**	**Area**	**±SD**	**Area Pred.**	**±SD**
**D3**	24225	3326	23475	4052	17986	1304	14047	11778
**L3**	25819	2791	22775	3609	2795	304	1500	10488
**D4**	40009	2593	40242	6768	25900	1068	14299	19667
**L4**	31807	3398	31277	6048	11886	351	14799	17575
**D5**	39584	5264	40796	7116	32985	105	32215	20679
**M4Q**	11834	2897	19058	6078	14146	43	16352	17662
**L5**	40281	7298	40503	8413	35961	754	43107	24448
**D6**	32088	6804	34212	5607	34523	1809	39860	16294

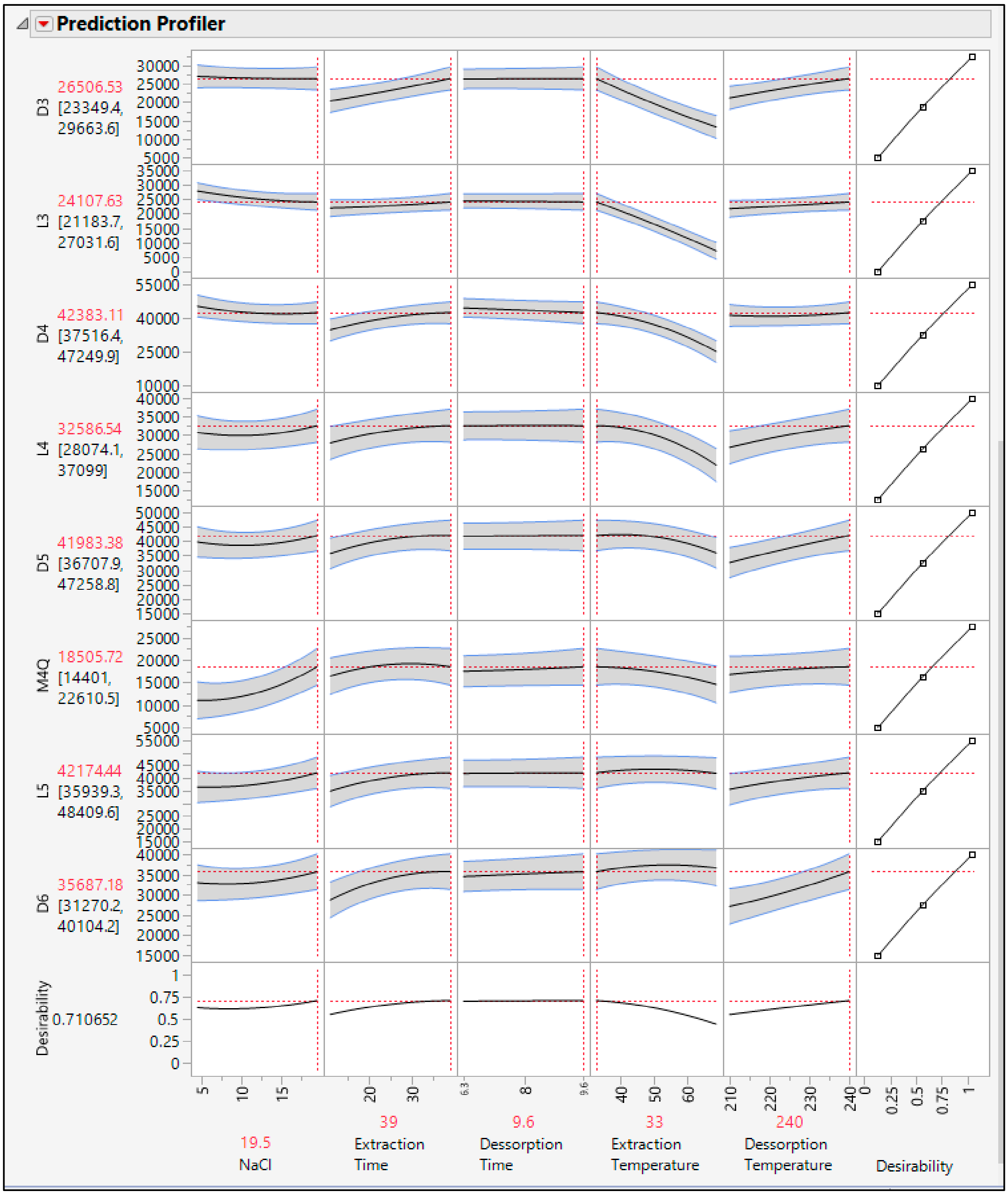

Figure S1. Optimal conditions and desirability function

(A)

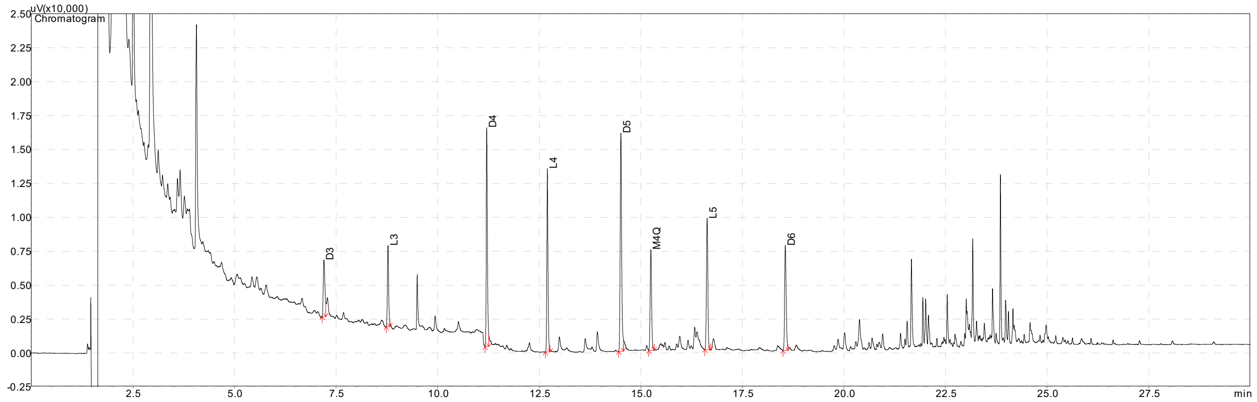

(B)

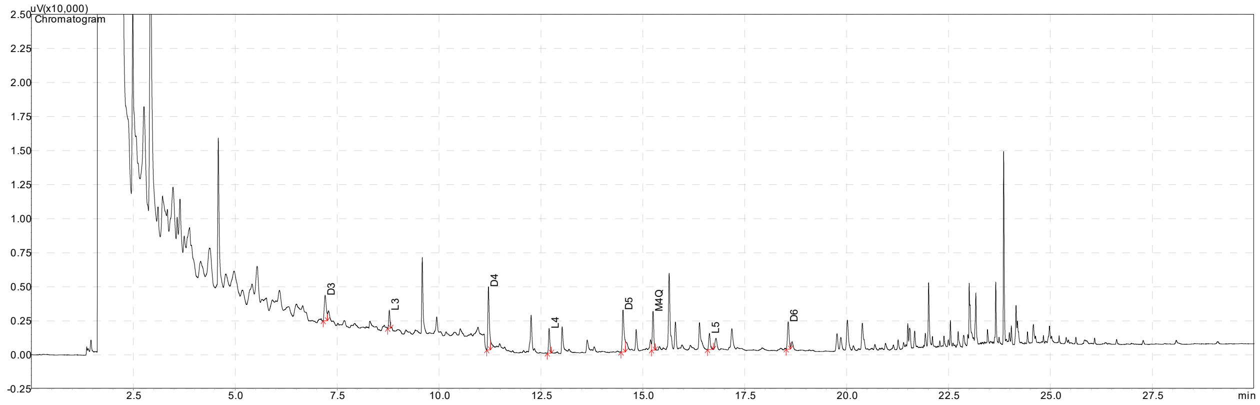

Figure S2. Chromatogram of a 3 µg L^{-1} calibration standard (A) and wastewater sample (B).
